# Embryo Buoyancy and Cell Death Gene Expression During Embryogenesis of Yellow-Tail Kingfish *Seriola lalandi*

**DOI:** 10.3389/fcell.2021.630947

**Published:** 2021-03-18

**Authors:** Jaime Palomino, Camila Gómez, María Teresa Otarola, Phillip Dettleff, Daniel Patiño-García, Renan Orellana, Ricardo D. Moreno

**Affiliations:** ^1^Departamento de Ciencias Fisiológicas, Facultad de Ciencias Biológicas, Pontificia Universidad Católica de Chile, Santiago, Chile; ^2^Laboratorio de Reproducción Animal, Facultad de Ciencias Veterinarias y Pecuarias, Universidad de Chile, Santiago, Chile; ^3^Laboratorio FAVET-INBIOGEN, Facultad de Ciencias Veterinarias y Pecuarias, Universidad de Chile, Santiago, Chile; ^4^Facultad de Medicina Veterinaria y Agronomía, Universidad de Las Américas, Sede La Florida, Santiago, Chile; ^5^Departamento de Ciencias Quiímicas y Biológicas, Facultad de Salud, Universidad Bernardo O’Higgins, Santiago, Chile

**Keywords:** *Seriola*, yellow-tail kingfish, apoptosis, buoyancy, pelagic

## Abstract

In pelagic fish, embryo buoyancy is a noteworthy aspect of the reproductive strategy, and is associated with overall quality, survival, and further developmental success. In captivity, the loss of buoyancy of early embryos correlates with high mortality that might be related to massive cell death. Therefore, the aim of this study was to evaluate under captivity conditions the expression of genes related to the apoptosis process during the early embryonic development of the pelagic fish *Seriola lalandi*, and its relationship to the buoyancy of embryos. The relative expression of *bcl2*, *bax-like*, *casp9*, *casp8*, and *casp3* was evaluated by RT-qPCR and FasL/Fas protein levels by western blot in five development stages of embryos sorted as floating or low-floating. All genes examined were expressed in both floating and low-floating embryos up to 24 h of development. Expression of the pro-apoptotic factors *bax, casp9, casp8*, and *casp3* was higher in low-floating as compared with floating embryos in a developmental stage-specific manner. In contrast, there was no difference in expression of *bcl2* between floating and low-floating embryos. Fas protein was detected as a single band in floating embryos without changes in expression throughout development; however, in low-floating embryos, three higher intensity reactive bands were detected in the 24-h embryos. Interestingly, FasL was only detected at 24-h in floating embryos, whereas in low-floating samples this ligand was present at all stages, with a sharp increase as development progressed. Cell death, as evaluated by the terminal deoxynucleotidyl transferase-mediated dUTP nick-end labeling assay, was highly increased in low-floating embryos as compared to floating embryos throughout all developmental stages, with the highest levels observed during the gastrula stage and at 24 h. The results of this study suggest that an increase in cell death, probably associated with the intrinsic and extrinsic apoptosis pathways, is present in low-floating embryos that might explain their lower developmental potential under captivity conditions.

## Introduction

*Seriola lalandi* is a globally distributed marine pelagic fish with growing importance worldwide for the aquaculture industry (Stuart and Drawbridge, 2013; Orellana et al., 2014; Sanchís-Benlloch et al., 2017). However, there are major knowledge gaps regarding its reproductive physiology and early embryo development that hamper scaling of production (Moran et al., 2007; Yang et al., 2016). A major distinctive reproductive strategy of pelagic fishes is the floatability (buoyancy) of eggs and embryos during the early stages of development. During captivity, the loss of buoyancy (i.e., sinking down to the bottom of the tank) of eggs and early embryos has been proposed as a major cause of developmental failure (Carnevali et al., 2001a; Sawaguchi et al., 2006; Sundby and Kristiansen, 2015). Buoyancy in pelagic fish embryos is a consequence of hydration that occurs mainly in the final stages of oocyte maturation in the ovary. This process involves passive water uptake by the oocyte, stimulated by an osmotic gradient created by accumulation of free amino acids derived from proteolysis of yolk proteins (Carnevali et al., 2001b; Fabra et al., 2005; Sawaguchi et al., 2006). There is evidence that the efficiency of the hydration process is highly relevant to the attainment of high buoyancy, allowing eggs and embryos to survive, and complete development (Ådlandsvik et al., 2001; Seoka et al., 2003; Jung et al., 2012, 2014). In addition, low embryo survival has been mentioned as one of the main drawbacks of pelagic fish farming, which is partly due to the production of low buoyancy eggs and embryos (Carnevali et al., 2001a, 2003; Thomé et al., 2012). Mechanisms associated with the cell death process, which would be determined during the formation of the oocyte in the ovary, have previously been associated with the loss of buoyancy in pelagic fish embryos (Carnevali et al., 2003; Thomé et al., 2012).

Regulated cell death involves a growing number of mechanisms that relay the cellular context and various stimuli (external or internal) to eliminate damaged cells or tissue remodeling during morphogenesis (Cole and Ross, 2001). Apoptosis is a particular and conserved, highly regulated type of cell death that is protein-directed, and necessary for the elimination of unwanted or altered cells, particularly during embryo development (Huang et al., 2000; Tittel and Steller, 2000; Bedoui et al., 2020). One of the main characteristics of apoptosis is the activation of a series of cysteine-aspartic intracellular proteases known as caspases (D’Arcy, 2019). The common strategy is to first assemble a multiprotein complex containing apical or initiator procaspases, which in turn activate executioner caspases (Shi, 2002, 2006). Apical caspase activation can be induced by the intrinsic (also known as the mitochondrial) pathway or the extrinsic (cell death receptor) pathway (Moreno et al., 2011; Kiraz et al., 2016; Bedoui et al., 2020). The extrinsic pathway begins with the activation of death receptors, such as tumor necrosis factor receptor (TNFR) or Fas (CD95/Apo-1) (Dewson and Kluck, 2009; Moreno et al., 2011), by binding with their cognate ligands (TNF-alpha and Fas ligand (FasL), respectively) (Kiraz et al., 2016). Activation of cell death receptors requires their trimerization and the formation of a multiprotein complex at the intracellular domain composed of inactive (pro-) apical initiator caspases (8 or 10), along with adaptor proteins such as FADD (Kiraz et al., 2016). Oligomerization of initiator caspases 8 or 10 induces activation, which is further stabilized by processing the prodomain, and proteolytic activation of executioner caspases 3 or 7 (Cullen and Martin, 2009; D’Amelio et al., 2010). In contrast, the intrinsic apoptotic pathway is initiated in response to a variety of stimuli that act on multiple targets within the cell (e.g., oxidative stress or DNA damage.). The main characteristic of this pathway is the complex interplay of pro-and antiapoptotic proteins of the BCL2 family that mainly regulate mitochondrial outer membrane permeability. In this way, BAK and BAX proapoptotic proteins play a major role in forming a pore at the mitochondrial outer membrane that allows the release of apoptogenic factors, including cytochrome *c* and DIABLO (also known as SMAC). This event allows the assembly of a multiprotein complex named the apoptosome, which is composed of apoptosis protease-activating factor-1 (APAF-1), cytochrome c, dATP, and the apical procaspase 9 (Willis and Adams, 2005; Youle and Strasser, 2008). Upon assembly into this complex, procaspase 9 becomes activated and then proteolytically activates the executioner caspases 3 and 7 (D’Amelio et al., 2010; Bedoui et al., 2020). Therefore, both pathways meet at the final caspase activation step (caspase3), which proteolyzes different cellular components, such as the inhibitor of calcium activated DNAase (ICAD), allowing release of CAD, an enzyme that degrades DNA internucleosomally (Luthi and Martin, 2007; Dix et al., 2008; Kiraz et al., 2016; Bedoui et al., 2020).

In fish, cell death as evaluated by DNA fragmentation or the presence of active caspase-3 has been shown to occur in the ovary as an important factor in progression of follicular atresia (Takle and Andersen, 2007; Thomé et al., 2012; Morais et al., 2016). In the tropical fish *Prochilodus argenteus*, high activity of caspase 3 and a greater number of apoptotic cells as revealed by means of the terminal deoxynucleotide transferase-mediated dUTP nick-end labeling (TUNEL) technique was observed in the ovarian cells of fish exposed to low oxygen concentrations. These observations coincided with the arrest and failure in the folliculogenesis process (Thomé et al., 2012). In this same species, BAX protein displayed an intense expression in follicles 2–3 days after spawning. In contrast, BCL2 showed higher expression immediately after spawning, and decreased on day 2 and 3 after spawning (Morais et al., 2016). Studies regarding the importance of apoptosis during fish embryonic development have been addressed mainly in zebrafish (Eimon and Ashkenazi, 2010) as a model to study teleost fishes; however, it is important to note that several steps in the reproductive strategy of marine pelagic species differ. In zebrafish, the spatial and temporal patterns of apoptosis during development, as evaluated by TUNEL labeling, shows an upregulation of cell death during the period 12–96 h post-fertilization (Cole and Ross, 2001). In transgenic zebrafish embryos overexpressing caspase 3, high levels of apoptosis and morphological abnormalities in specific tissues were detected. These embryos were also more sensitive to ultraviolet radiation compared to wild type (Yamashita et al., 2008). Similar findings were reported when mRNA encoding caspase 3 was injected into zebrafish early embryos (Valencia et al., 2007). Regarding participation of components of the extrinsic pathway of apoptosis during zebrafish embryogenesis, it was observed that overexpression of caspase 8 induced embryonic mortality (Eimon et al., 2006). Overall, these findings suggest that eggs and embryos of teleost fishes have the machinery to elicit cell death during normal development or after environmental injuries (AnvariFar et al., 2017).

To our knowledge, there is only one report addressing the relationship between apoptosis and the buoyancy levels of eggs in a pelagic teleost fish (Carnevali et al., 2003). In the gilthead seabream *Sparus aurata*, it was shown that non-floating eggs had a lower capacity for protein and RNA synthesis, which led to them being less active than floating eggs. Furthermore, non-floating eggs displayed apoptotic characteristics such as cellular contraction, DNA fragmentation and an increased volume of mitochondria (Carnevali et al., 2003). Additionally, participation of the Fas/FasL system was proposed, since both proteins were detected in non-floating eggs, but only Fas was present in floating ones (Carnevali et al., 2003). Therefore, the aim of this study was to determine whether the loss of buoyancy of *S. lalandi* embryos during early development is related to increased cell death and expression of a particular apoptosis pathway.

## Materials and Methods

### Ethical Approval

All procedures were reviewed and approved by the Ethic and Animal Care Committees of the Faculty of Biological Sciences of Pontifical Catholic University of Chile, Faculty of Veterinary Sciences, University of Chile and Research Ethics Committee of the Chilean National Foundation for Scientific and Technological Research.

### Animal Management and Sampling Procedures

**FIGURE 1:**
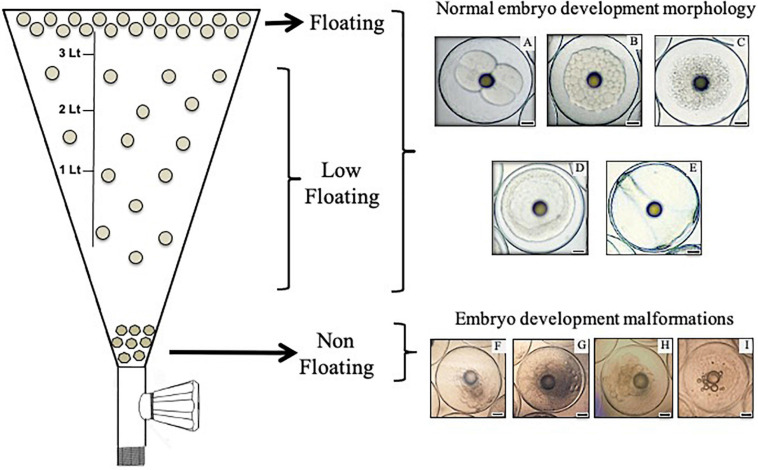
Scheme representing the process of separation and collection of embryos according to their buoyancy level in a 4 L inverted flask (left side). In this work, only floating and low-floating embryos were collected and assessed. Floating samples were collected from the surface of the flask. Using of a faucet located at the bottom of the flask, non-floating embryos were discarded and embryos displaying a reduced buoyancy level (classified here as low-floating) were obtained by extraction of 3 L of water. The right side shows images of representative embryos of the development stages collected in this work. **(A)** Considered first and second cleavage, **(B)** morula, **(C)** blastula, **(D)** gastrula and **(E)** 24 h post-fertilization embryos (2/4C, M, B, G, and 24H, respectively). Normal morphology was observed in both floating and low-floating embryos. **(F–I)** Embryo development malformations, which are representative of non-floating embryos. Bars = 0.2 mm.

Broodstock was comprised of 90 adult individuals conditioned in three indoor tanks 2.5 m deep and with a capacity of 20,000 L in the hatchery center of Acuinor SA Company, Caldera, Atacama Region, Chile. Animals were kept at a male to female ratio of 2:1 with artificial thermo-photoperiod management, where spawning events occurred spontaneously with temperatures above 19°C and 14 h of light. Feeding consisted of commercial pellets (Vitalis^®^, Skretting, Puerto Montt, Chile) and fresh food (fisheries fish, squid, and cuttlefish) that were provided according to the protocols of the company. Samples for this study were collected during three spawning periods (one season per tank between January 2017 and March 2018). Two independent batches from each tank (six biological replicates or six batches) were monitored from spawning, and embryos were collected at different developmental stages as described by Palomino et al. (2014, 2017). Briefly, fertilized eggs were channeled from a skimmer on the surface of each tank into a separate egg collector tank, where the embryo development progressed (22–23°C). The development stages collected in this work were 2/4C (considered first and second cleavage), morula, blastula, gastrula, and 24 h post fertilization embryos, which are identified as 2/4C, M, B, G, and 24H, respectively (Figures 1A–E). Figure 1 represents the separation procedure of embryos according to their buoyancy level (left side) and representative morphological characteristics of these embryos (right side). Embryos were transferred from the embryo collector tank to a conical 4 L inverted flask, where they were kept for 10 min. After this time, floating samples (approximately 50 individuals) were collected from the surface using a 500 μm mesh. Sinking embryos located at the bottom of the flask, which were dead and displayed structural damages as described in *S. aurata* by Carnevali et al. (2003), were discarded by removing 200 mL of water by means of a faucet. Embryos displaying a reduced buoyancy level (classified as low-floating) were obtained by extraction of 3 L of water where approximately 20 individuals could be collected. Samples were stored in RNAlater solution (Ambion^®^, Thermo Fisher Scientific, Waltham, MA, United States) for assessment of mRNA expression through real-time polymerase chain reaction (RT-qPCR) or frozen at −20°C for western blot analysis. For TUNEL assays, floating and low-floating embryos from M to 24H stages were processed for cell separation as described below. Ovary samples obtained by cannulation of the gonophore of three anesthetized adult females were used to standardize qPCR assay conditions.

### Batches and Sample Characterization

The hatching rate of each spawning batch used in this study was quantified after 70 h of incubation of embryos in the egg collector tank (22–23°C) using the morphological criteria described by Moran et al. (2007). Thus, the fraction of newly hatched larvae approximately 4.8 mm in length was registered in a 50 mL sub-sample. Buoyancy, evaluated as the floating rate (FR) at different developmental stages, was assayed in a 30 mL sub-sample obtained from the egg collector tank, which was deposited during 10 min in a 50 mL beaker. Then, floating samples were isolated and their fraction recorded (%FR) by counting both floating and low-floating embryos. Additionally, in order to ensure that data obtained in this work corresponded to the developmental stage proposed, a sample of 20 embryos was fixed in 4% formaldehyde for evaluation under phase contrast microscopy with a Leica DME microscope (Leica Microsystems, Wetzlar, Germany). Batches used met criteria for morphological homogeneity (Moran et al., 2007; Yang et al., 2016), where at least 70% of the individuals corresponded to the same stage of development (*n* = 14). Sample characterization also included the assessment of the diameters of individuals and their oil globules in floating and low-floating embryos at each developmental stage. An eyepiece graticule ocular lens was used for these determinations, which were performed at 100 × magnification with a Leica DME phase contrast microscope.

### RNA Isolation and Real-Time Polymerase Chain Reaction

One single total RNA extraction was done from each spawning event (six biological replicates). For this, Gene JET RNA Purification Kit (Thermo Scientific, Eugene, OR, United States) was used according to the manufacturer’s instructions. The concentration of total RNA was determined in a Qubit^®^ fluorometer using the Qubit^®^ RNA Assay Kit, (Molecular Probes Invitrogen, Eugene, OR, United States). DNA contamination was removed by DNase I treatment, and reverse transcription (RT) was performed using the SuperScript^TM^ First-Strand Synthesis System (Invitrogen). Then, complementary DNA (cDNA) concentration was determined using the ssDNA Qubit^®^ Assay Kit (Molecular Probes^®^, Invitrogen). The primers used in this study are shown in Table 1. Primers for target genes *bcl2, bax, casp9, casp3*, and *casp8* were designed using Primer3^1^ for amplicons between 80 and 213 base pairs (see Table 1). Gene amplifications were in performed in triplicate using an Illumina^®^ Eco Real-Time PCR System Model EC-100-1001 and Maxima SYBR Green/Fluorescein qPCR Master Mix (Thermo Fisher Scientific, Waltham, MA, United States) following the manufacturers’ conditions. Control samples without reverse transcriptase, cDNA template, and primers were included in each plate. Relative expression analysis for each target gene was performed in floating and low-floating embryos during different embryo development stages using the 2^–ΔΔCt^ method (Vandesompele et al., 2002). The constitutive genes *actb* and *map1b* were used as reference genes for normalization of expression (Palomino et al., 2017).

**TABLE 1:** Sequence of qPCR primers.

Gene	Entrez gene ID	Forward sequence (5′-3′)	Reverse sequence (5′-3′)	Size (pb)
*casp3*	111663309	CTTGTGGTTCACTCGTGTCA	ATACTATGACCGGGTCCTGG	104
*casp8*	111666542	CTGACACGAGGAGGAAGAGG	ATTGGGCAGAAGACAAATCG	213
*casp9*	111651602	TCCCTTTCAGGTGATGGACT	CTCTCCCAGTTTCCTCTCCA	106
*bax*	111669314	GGTGGAACAACTGCTCAAGA	TGCATGAAGATGTCCTGAGC	105
*bcl2*	111670485	CAAGGAGGAGATGACATCGC	TCCAGCTGTTAAGAGGTCCA	80
*actb*	111225231	AGGGAAATCGTGCGTGACAT	GCTGAAGTTGTTGGGCGTTT	563
*map1b*	111647584	TCATCAAGATTATCAGGAGGCG	GGAAGCATACACCATGTAGAGG	158

### Western Blot Analysis

Expression levels of FasL/Fas protein were determined using western blot analysis of floating or low-floating embryos at different developmental stages. Three protein extraction procedures were performed based on three pools, with each one constituted of embryos obtained from two independent spawning events (a total of six spawning events). Embryos were put into 1 mL of RIPA buffer (50 mM Tris–HCl, pH 8.0, 250 mM NaCl, 2 4 mM EDTA, and 1% NP-40) plus protease inhibitor cocktail (Pierce, Thermo Fisher Scientific). Samples were then sonicated for 30 s with 50% pulse in a Branson 5,450D Digital Sonifier, (Branson Ultrasonics, Danbury, CT, United States) and centrifuged at 14,000 × *g* for 15 min at 21°C to remove cellular debris. Protein concentrations were quantified using a Qubit 2.0 fluorometer (Invitrogen, Budapest, Hungary) and the Qubit Protein Assay Kit (Molecular Probes^®^, Invitrogen). Then, 50 μg of protein extract from each sample was resolved in a 12% SDS-polyacrylamide gel at 120 V in 1% running solution (TRIS 25 mM, glycine 250 mM, 0.1% SDS in distilled water). Electrophoretically separated peptides were transferred to a polyvinylidene-fluoride membrane (Immobilon-P membrane, Millipore Corp., Bedford, MA, United States) using the Mini-Vertical Slab Gel/Blotting Electrophoresis System (DCX-700, C.B.S. Scientific Company, Inc., Del Mar, CA, United States). The membranes were immersed in blocking solution (100 mM Tris–HCl, pH 8.0; 5% (w/v) skim milk; 35 mM NaCl and 0.1% (v/v) Tween 20 (Sigma-Aldrich, St Louis, MO, United States) for 30 min and incubated overnight at 4°C with an antibody previously tested in fish by Carnevali et al., 2003 (rabbit anti-mouse Fas polyclonal antibody diluted 1:200, Santa Cruz Biotechnology Cat# sc-1023, RRID:AB_2100247). Its specificity was ascertained using a blocking peptide (Santa Cruz Biotechnology Cat# sc-716, RRID:AB_2100358). To detect Fas ligand/TNFSF6, we used a mouse anti-rat monoclonal antibody diluted 1:100 (R and D Systems Cat# MAB1858, RRID:AB_2100788), which results in a band ≈44 kDa consistent with membrane-Fas ligand/TNFSF6 as revealed by Carnevali et al. (2003) using a Santa Cruz Biotechnology antibody. After washing with 0.1% phosphate buffered saline (PBS)-Tween 20, membranes were incubated for 1 h at 20–23°C with 1:500 of goat anti-rabbit antibody (Abcam Cat# ab6722, RRID:AB_954595) or goat anti-mouse IgG antibody (Abcam Cat# ab6790, RRID:AB_954670) conjugated to alkaline phosphatase. Charge controls were performed by incubating the membranes with 1:500 of anti-β-actin antibody (Sigma-Aldrich Cat# A1978, RRID:AB_476692), and incubation with the second antibody was performed as a negative control. Protein bands were visualized with 5-bromo-4-chloro-3-indolyl phosphate (BCIP)/nitro blue tetrazolium (NBT) substrate (SC-24981, Santa Cruz Biotechnology Inc.) in 0.1 M Tris, pH 9.5. Band intensity was evaluated by densitometry using the Gel Documentation System Biosens SC-645 (Biotop, Shanghai Bio-Tech Co., Shanghai, China) and expressed as arbitrary units of target protein/β-actin ratio.

### Cells Preparation for TUNEL Assay and Flow Cytometry Assessment

In order to identify the fragmented DNA that is a hallmark of apoptotic cells in floating and low-floating *S. lalandi* embryos, the TUNEL method was applied through flow cytometry in cell suspensions obtained from morula to 24H embryos. Cell suspensions were prepared according to the embryo dissociation method described by Bresciani et al. (2018). Briefly, approximately 30 embryos were mechanically dissociated by harsh pipetting in 500 μL of a dissociation mix solution of 460 μL of 0.25% trypsin-EDTA (Gibco Life Technologies, Carlsbad, CA, United States) plus 40 μL of 100 mg/mL of collagenase (Sigma-Aldrich). This procedure was performed in a 1.5 mL Eppendorf tube over a heat block set at 30°C until the sample was fully homogenized. The dissociation procedure was stopped by adding 800 μL of Dulbecco’s modified Eagle’s medium (Merck KGaA, Darmstadt, Germany) supplemented with 10% fetal bovine serum (Merck KGaA). This mixture was centrifuged for 5 min at 700 × *g* and the supernatant discarded. Pelleted cells were washed twice by resuspension in 500 μL of 1 × PBS and fixed in 2% paraformaldehyde in PBS. Then, pelleted cells were washed twice in 1 × PBS by centrifugation and permeabilized by incubation in 100 μL of 0.1% Triton X-100 prepared in 0.1% sodium citrate for 2 min on ice. After washing twice in 1 × PBS, cells were treated with the *In Situ* Cell Death Detection Kit, Fluorescein (Roche Diagnostics Gmbh, Mannheim, Germany) according to the company’s protocol for cell suspensions. Before analysis in the flow cytometer, cells were stained with 1 mg/mL propidium iodide (PI) to discriminate cells from debris. The TUNEL reaction was assessed though flow cytometric analyses on a Gallius Cytometer (Beckman Coulter, Brea, CA, United States) with standard settings, including compensation protocols for proper fluorophore discrimination. Events with very low forward scatter, representing debris, were excluded from analysis by forward and side-scatter gating. The proportion of labeled cells with PI and positive for TUNEL reaction were detected on a logarithmic scale in the FL3 and FL1 fluorescence detectors, respectively.

### Statistical Analysis

Six replicates, which are representative of different batches of separate spawning events, were used for morphometric evaluations (buoyancy rate, embryo and oil drop diameters), analysis of gene expression, and flow cytometry assessments. For western blots and band intensity analysis, three replicates were made. Data were analyzed by ANOVA using the InfoStat Professional Program, National University of Córdoba, Argentina. Each model included the main effects of the developmental stages, the buoyancy conditions and their interaction. Significant differences among means were evaluated using Tukey’s test. All values were considered significantly different for *P* < 0.05 (Sokal, 1995).

## Results

### Characterization of Batches and Samples

**FIGURE 2:**
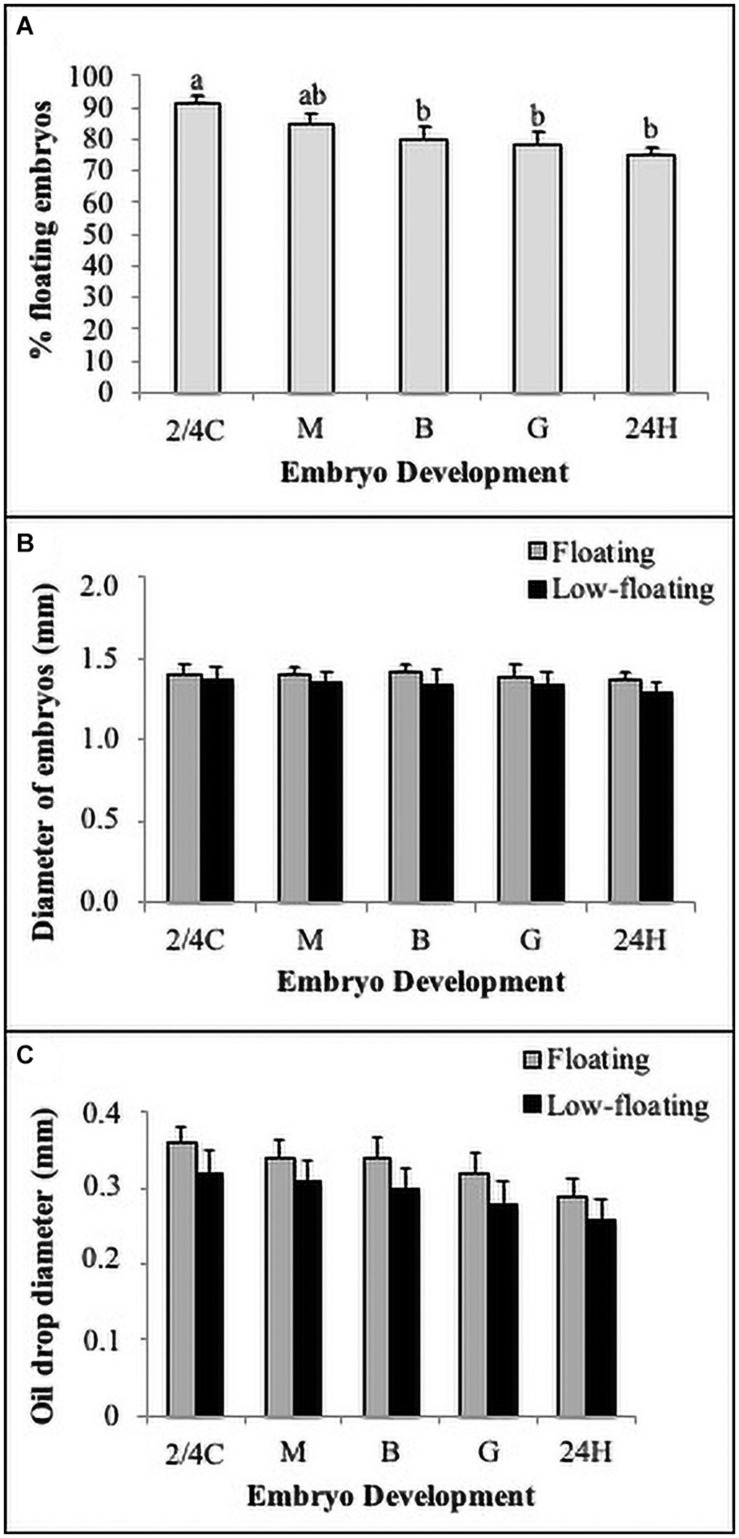
Morpho-biometric parameters of embryos. **(A)** Buoyancy level (% floatability) of batches observed in each developmental stage. **(B)** Total embryo and **(C)** oil drop diameters quantified in floating (gray bars) and non-floating (black bars) embryos. Bars represent mean ± SD of six replicates and the different letters above the bars indicate significant differences (*P* < 0.05). *N* = 120.

The six batches assessed in this work displayed an average hatching rate of 85.5 ± 7.5% [mean ± standard deviation (SD)] after 70 h of incubation. As stated in the introduction, as part of *S. lalandi* reproductive strategy, the developmental competence of eggs and embryos is related to the buoyancy level at the different early developmental stages. The embryo stages collected in this work were first and second cleavage (2/4C), morula (M), blastula (B), gastrula (G), and 24 h post fertilization embryos (24H) (Figures 1A–E). Embryos were divided into three different categories according to buoyancy level: (1) floating samples were embryos that remained at the top of the water column and showed translucid and clear blastomeres (Figures 1A–E); (2) low-floating samples were embryos that had a lower buoyancy and remained in a middle range between the surface and the bottom of the tank; they showed a similar aspect to the floating sample; and (3) non-floating embryos were samples that sank to the bottom of the tank. They usually showed fragmented and opaque blastomeres (Figures 1F–I). These embryos did not reach further developmental stages. Given that non-floating embryos showed morphological characteristics of cell death and have been previously characterized in pelagic eggs (Carnevali et al., 2003), we compared the floating versus the low-floating populations.

In the present study, most of the embryos retained a high buoyancy (>80%) at all developmental stages; however, embryos beyond gastrula stages showed a significant reduction in floatability (Figure 2A). When analyzed separately, embryo diameter was similar between floating and low-floating samples, regardless of developmental stage (Figure 2B). Another morphological parameter frequently used in fish embryo/egg analysis is the diameter of cytoplasmic oil drops, which is regarded as an important element to confer buoyancy during the first stages of development. Results showed that oil droplet diameter did not change throughout development or when compared between floating and low-floating samples, suggesting that other mechanisms influence the loss of buoyancy (Figure 2C).

### Detection of Apoptosis Elements in *Seriola lalandi* Embryos

**FIGURE 3:**
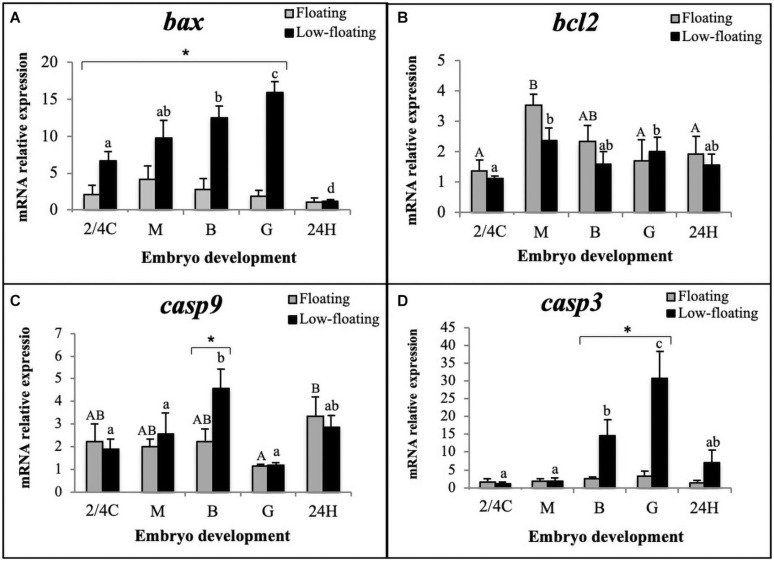
Relative expression assessment of **(A)** *bax*, **(B)** *bcl2*, **(C)** *casp9*, and **(D)** *casp3* mRNAs throughout *S. lalandi* embryo development in floating (gray bars) and low-floating (black bars) samples. Different uppercase letters above bars indicate statistical differences within floating embryos. Similarly, different lowercase letters above bars indicate statistical differences within low-floating embryos. Differences between floating and low-floating embryos are identified with an asterisk over a bracket. Bars represent mean ± SD of six replicates. Results were considered statistically significant when *P* < 0.05.

Levels of *bax* mRNA encoding the proapoptotic BAX protein remained similar throughout all developmental stages in floating embryos (Figure 3A). In low-floating embryos, there was a constant increase in levels of *bax* mRNA from 2/4C to G stage, and expression was sharply higher than in floating samples (Figure3A, asterisk). Interestingly, *bax* mRNA levels were similar between floating and non-floating embryos at the 24H stage (Figure 3A). In contrast, *bcl2* mRNA levels were similar between floating and non-floating embryos at all developmental stages, and showed a sharp increase from 2/4C to M in both groups and remained similar up to 24H only in low-floating embryos (Figure 3B). The levels of *bcl2* mRNA decreased at G and 24H only in floating embryos (Figure 3B).

Since upregulation of *bax* and downregulation of *bcl2* is related to activation of the intrinsic apoptotic pathway, we examined the mRNA levels of *casp9*, which encodes the initiator caspase in this pathway. Our results showed that *casp9* mRNA levels significantly increased in low-floating embryos when they reached the B stage and then decreased at G (Figure 3C). Only B stage low-floating embryos had a higher level of *casp9* mRNA expression than floating samples. Interestingly, in floating samples, levels of *casp9* were higher at 24H than G. Executioner *casp3* mRNA levels were similar between floating and low-floating samples at 2/4C, M, and 24H stages but were significantly higher in low-floating embryos at B and G stages (Figure 3D). These results suggest that the intrinsic pathway is active in low-floating embryos.

**FIGURE 4:**
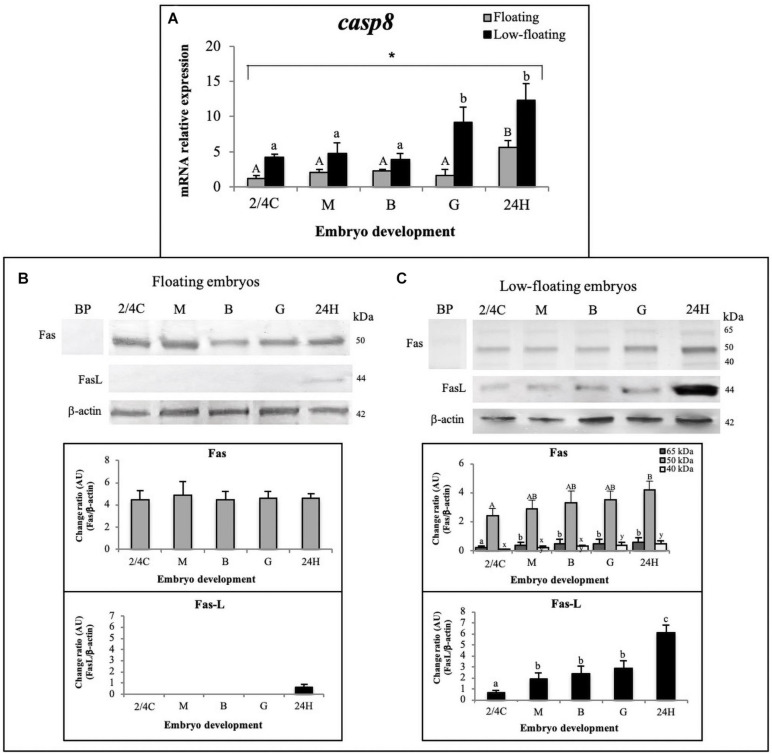
Detection of the apoptosis extrinsic pathway throughout *S. lalandi* development in floating and low-floating embryos. **(A)** Relative expression assessment of *casp8* mRNA, in floating (gray bars) and low-floating (black bars) samples representing mean ± SD of six replicates for floating and low-floating embryos, respectively. Different uppercase or lowercase letters above bars indicate statistical differences (*P* < 0.05) within floating or low-floating embryos, respectively. Differences between floating and low-floating embryos are identified with an asterisk over a bracket. **(B,C)** Western blot analysis of Fas and FasL proteins during *S. lalandi* embryo development in floating **(B)** and low-floating **(C)** embryos. In floating embryos **(B)**, both Fas and FasL were detected as single bands of 50 and 44 kDa, respectively. However, in low-floating embryos **(C)**, western blot analysis of Fas revealed three bands of 65 (dark gray bars), 50 (gray bars) and 44 (white bars) kDa. In these samples, FasL was detected as a single band of 44 kDa. Bands were analyzed by densitometry. BP: Antibody anti-Fas preincubated with a blocking peptide. Bars represent the mean ± SD intensity of each band. Different uppercase letters above the bars indicate statistical differences within floating embryos. Similarly, different lowercase letters above bars indicate statistical differences within low-floating embryos (*P* < 0.05).

Concerning the extrinsic apoptotic pathway, the mRNA levels of *casp8* in low-floating embryos were higher than in floating samples regardless of developmental stage, and reached their highest levels at the G stage in low-floating embryos and then at 24H in floating and low-floating samples (Figure 4A). We next sought to determine at the protein level if *S. lalandi* embryos express Fas and FasL, the initiator of the extrinsic pathway. In floating samples, Fas was detected as a single band of 50 kDa, with similar expression levels throughout all the studied stages of development (Figure 4B). In low-floating embryos, the antibody against Fas detected a strong band of 50 kDa and two other fainter bands of 65 and 40 kDa. The 50 kDa band detected here with the antibody against Fas is similar to that reported in mammals. The three bands (65, 50, and 40 kDa) detected in low-floating embryos were not observed when the antibody against Fas was pre-incubated with a blocking peptide, suggesting that the label was specific (Figures 4B,C). Interestingly, in floating eggs, the ligand of Fas (FasL) was detected as a single weak band of 44 kDa only in 24H embryos (Figure 4B). In contrast, in low floating eggs, the antibody against FasL gave a single faint band of 44 kDa in 2/4C embryos, then increased at M stage. It remained steady until G stage and then increased again in 24H embryos (Figure 4C). These results suggest the presence of the extrinsic pathway components in *S. lalandi* during early development.

**FIGURE 5:**
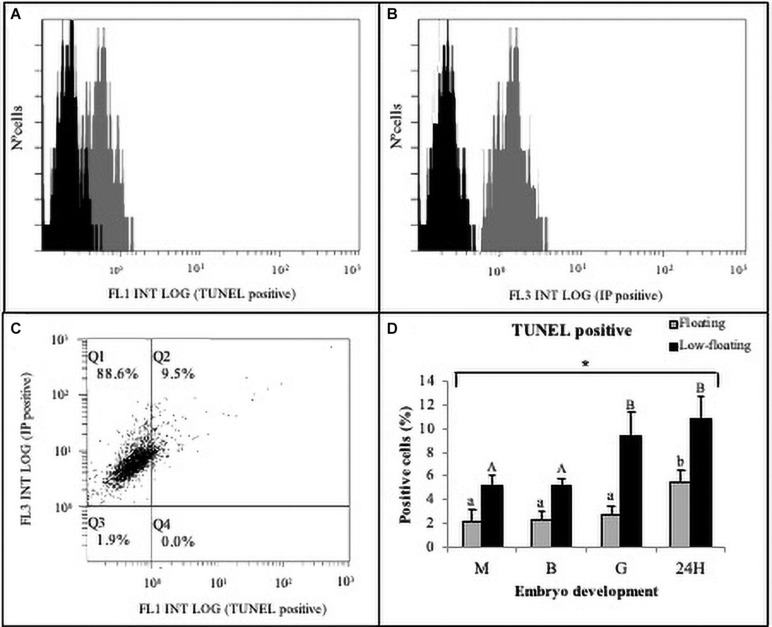
Cell death detection in *S. lalandi* early development. Floating and low-floating embryos cells were disaggregated at each developmental stage as described in the materials and methods. Cell death was assayed by terminal deoxynucleotide transferase-mediated dUTP nick-end labeling (TUNEL) in cell suspensions using flow cytometry. **(A,B)** Representative histograms resulting from flow cytometry analysis to TUNEL **(A)** and propidium iodide (PI) uptake **(B)**, which were detected by FL1 and FL3 detectors, respectively. The horizontal axis of the histograms represents the signal intensity (log scale) and the vertical axis represents the number of cells. Each histogram displays the populations of both negative control cells (black curve) and cells stained by TUNEL and PI (dark gray curves). No signal was detected with a negative control of autofluorescence, and cells were detected within the first decade of logarithmic scale for both stains. **(C)** Representative fluorescence dot plot analysis of embryo cells positive to TUNEL staining (FL1) versus PI (FL3). Quadrants Q1 and Q2 represent PI positive cells, and debris were discarded in quadrants Q3 and Q4. TUNEL positive cells were detected in quadrant Q2. Cells used for flow cytometer representations **(A–C)** correspond to low-floating gastrulae embryos. **(D)** Bar graph summarizing the analysis by flow cytometry of TUNEL staining in cells obtained from different embryonic stages in floating (gray bars) and low-floating (black bars) samples. Bars represent the mean ± SD of six experimental replicates and 2,000–4,000 cells were evaluated in each experiment. Different uppercase letters above the bars indicate statistical differences within floating embryos. Conversely, different lowercase letters above bars indicate statistical differences within low-floating embryos. In all stages assessed, there were statistical differences between floating and low-floating embryos, which are identified with an asterisk over a bracket. Differences were considered statistically significant when *P* < 0.05.

Finally, in order to determine the extent of cell death, blastomeres of embryos at different developmental stages were disaggregated, and DNA fragmentation was evaluated using TUNEL. A representative result from low-floating G embryos shows the evaluation of TUNEL (+) cells (Figure 5A) and propidium iodide uptake as a measure for cellular debris (Figure 5B). Dot plot analysis showed the Q1 and Q2 populations that were used in the analysis (Figure 5C). Results indicated that M, B, G, and 24H low-floating embryos showed a higher level of TUNEL (+) cells than floating embryos, regardless of the developmental stage (Figure 5D). Cell death was similar in between M and B, regardless of their buoyancy state, and sharply increased at G stage only in low-floating samples. Floating 24H stage embryos showed a sharp increase in TUNEL labeling as compared with the other stages (Figure 5D).

## Discussion

Under captive conditions, it has been observed that the developmental potential of *S. lalandi* embryos rests largely on their buoyancy (Moran et al., 2007; Yang et al., 2016). Buoyancy is a characteristic of many pelagic marine fishes and is associated with embryo survival and development. In this work, we have identified a differential expression of apoptosis markers in embryos with low buoyancy as compared to those with positive buoyancy, suggesting a close association between cell death and loss of buoyancy during early development.

In marine pelagic species, the loss of buoyancy in eggs and embryos has been shown to be associated with a decrease or failure in development. Various morphological abnormalities and characteristics related to cell death have been observed in non-floating spawned eggs/embryos (i.e., those that sink to the bottom of the culture tank) of *S. aurata* under captive conditions (Carnevali et al., 2001a, 2003). In the current study, we observed that non-floating embryos showed several morphological malformations in the blastomeres and cytoplasmic oil droplets. These malformations clearly preclude further development, so we devoted our study to comparing the expression of molecules associated with apoptosis in embryos that remain on the surface (considered viable and good quality) to those that although remaining floating, did do so to a lesser degree. No differences between floating and low-floating embryos were found in the diameter of the embryo or in the oil drop at any of the observed stages. In addition, morphologic differences were not evident between these samples. Considering that only embryos with high buoyancy are completely viable for complete development, these results indicate that using embryo morphology alone as a quality marker is not a good parameter for assessing viability and development potential.

Embryos with low buoyancy are frequently observed in the culture of pelagic species, even in spawning lots that present high survival parameters. Among these parameters, the hatching rate obtained in this work (85%), was higher than that seen in other pelagic fish, such as the common dentex (*Dentex dentex*), where a hatching rate of 61.6% was observed (Samaee et al., 2010) and the medregal *Seriola dumerili*, with a hatching rate of 65% (Jerez et al., 2006). However, a decrease in the proportion of floating embryos was observed as development progressed. This decrease in buoyancy throughout development has been documented in others marine pelagic species, such as the Atlantic cod *Gadus morhua* (Jung et al., 2012, 2014), the European anchovy *Engraulis encrancicolus* (Ospina-Álvarez et al., 2012) and the blue whiting *Micromesistius poutassou* (Ådlandsvik et al., 2001). In these species, the loss of buoyancy throughout development was associated with a slight increase in the specific gravity of the embryo, which has been attributed mainly to the passive loss of water to the hyperosmotic environment until the formation of osmoregulation mechanisms, which occurs after gastrulation in teleost fish (Riis-Vestergaard, 1987; Pérez-Robles et al., 2015). Therefore, in this work we attempted to determine whether the physiological processes of buoyancy are related to programmed cell death.

Here, we show in both floating and low-floating eggs the presence of a major band with a size of 50 kDa that was detected with an anti-Fas antibody. Interestingly, in low-floating eggs alone we detect two other higher and lower molecular weight bands. In mammals, it has been reported that Fas consists of 335 aa and a size of 35 kDa (Takahashi et al., 1994; Peter et al., 2003). Since this protein is *N*-glycosylated at two sites in the extracellular domains, in addition to undergoing other post-translational modifications such as phosphorylation, palmitoylation, nitrosylation and glutathionylation, the mature form ranges between 48–54 kDa, which is close to the size of 50 kDa reported in the present work (Peter et al., 2003; Seyrek and Lavrik, 2019). The lower and higher molecular weight bands may represent different forms of post-translational modifications. This hypothesis is reinforced by the finding that when using the blocking peptide, all three bands detected by the antibody were no longer observed.

The FasL antibody detected only one band of 44 kDa in floating and low-floating samples. Interestingly, the genome of *Seriola dumerili* (greater amberjack), a close related species, shows three FasL-like genes with a predicted range of 238–267 aa (REF), which is very close to the 278 aa mouse protein (Suda et al., 1993). The predicted size of FasL is 32 kDa, but since it has several glycosylation sites, the mature form ranges between 38–42 kDa, which is close to the 44 kDa protein detected in the present study (Suda et al., 1993; Peter et al., 2003).

We observed that embryos with low buoyancy had higher levels of various apoptosis markers compared to high buoyancy embryos. Furthermore, we did not observe significant variations in cell death markers in floating embryos in the stages studied, except for an increase in *Casp9*, *Casp8*, and FasL in the 24H embryos. These results suggest that there is no significant morphological remodeling due to apoptosis during early development in *S. lalandi* until the 24H stage. However, further studies using higher resolution techniques are needed to determine whether local increases of cell death that were not resolved in this study are important during early development. Taking into account that the floating embryos represent normality in our model, the results of this work agree with evidence that relates apoptosis with normal development of the notochord in vertebrates. In zebrafish, it has been shown that the notochord begins to form during gastrulation, and that the extrinsic apoptotic pathway mediated by the Fas/FasL system is involved in the regression of notochord cells for their correct formation (Ferrari et al., 2014). Considering the developmental stages evaluated in this work, the formation of the notochord in the development of *S. lalandi* would occur between the gastrula stages and 24-h embryos, so the increase in the expression of apoptotic markers of the extrinsic pathway is consistent in these embryos. The results suggest that components of both the intrinsic and extrinsic pathways of apoptosis are expressed in the early development of *S. lalandi*.

**FIGURE 6:**
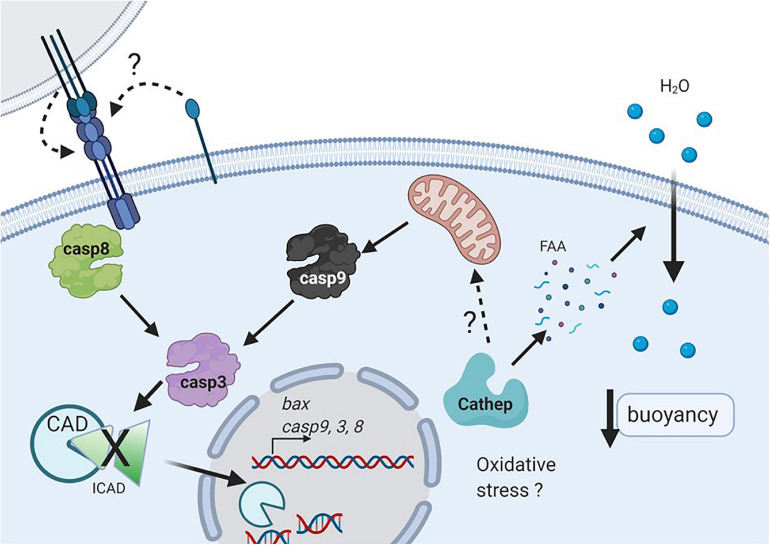
Working model of cell death during early development in pelagic fish. Oxidative stress or nutrient deprivation (e.g., autophagy) may activate cathepsins (probably cathepsin D), that could degrade intracellular yolk proteins and then increase free amino acids (FAA), and promote water uptake which determines egg and embryo buoyancy. Cathepsin D may activate the intrinsic pathway (e.g., caspase 9), which along with caspase 8 will activate the executioner caspase 3 (Figure 6). In addition, the extrinsic pathway may be triggered by activation of Fas upon autocrine or juxtacrine binding of FasL. Active caspase 3 will degrade different substrates such as ICAD, allowing the activation and translocation of CAD to the nucleus, where it can degrade DNA (Created with BioRender.com).

Previous studies in *S. aurata* (Carnevali et al., 2001a, 2003) showed expression of both Fas and FasL in non-floating unfertilized eggs, whereas only Fas was expressed in floating eggs. These results agree with the expression profile of these proteins observed in the present study at early stages of development (from 2/4C to gastrula) in low-floating embryos, suggesting that upregulation of FasL might promote or be involved in cell death. FasL-induced Fas activation in low-floating embryos may be either autocrine or paracrine (Figure 6), leading to induction of the extrinsic pathway as suggested by our results showing a high expression level of *casp8* mRNA at all developmental stages, and *casp3* mRNA in B and G stages.

The intrinsic pathway is initiated by mitochondrial damage, which has been reported in non-floating eggs of *S. aurata* eggs (Carnevali et al., 2003). Furthermore, a greater amount of protein was detected in floating compared with non-floating eggs in *S. aurata*. It was hypothesized that the higher protein availability in floating eggs could generate a higher amount of free amino acids resulting from their proteolytic processing, which would increase the internal osmotic strength, stimulating egg hydration (passive water uptake) and increasing the buoyancy of eggs and embryos (Carnevali et al., 2001a; Fabra et al., 2005). In addition, the authors observed lower levels of cathepsin D and L activity in sinking eggs as compared to floating ones. Cathepsins are the main enzymes involved in yolk proteolysis, resulting in free amino acids (Carnevali et al., 2006; Sawaguchi et al., 2006; Palomino et al., 2017). The floating eggs of *S. aurata* presented high activity of both cathepsins, but in the non-floating eggs, very high levels of cathepsin D and very low levels of cathepsin L were present (Carnevali et al., 2001a). There is evidence that cathepsin D induces apoptosis both *in vitro* and *in vivo* and promotes the release of cytochrome C from the mitochondria (Chwieralski et al., 2006; Minarowska et al., 2007). Therefore, one of the possible physiological upstream signals of apoptosis induction in eggs of pelagic fish could be the loss of buoyancy followed by activation of cathepsin D (Figure 6). In previous studies in *S. lalandi*, a similar pattern of enzymatic activity in floating eggs to that described in *S. aurata* was observed (Palomino et al., 2017); therefore, it is possible that given the similarities in the reproductive strategy of both species, the molecular characteristics related to apoptosis seen in this work in embryos of low buoyancy are also related to the participation of cathepsin D in the induction of apoptosis (Figure 6).

In addition, one of the interesting results was that embryos with low floatability showed a higher rate of cell death than the floating ones, even though the morphology between both groups was not different. In this work, cell death was evaluated using the TUNEL technique, which is a tool that allows the extent of DNA fragmentation to be determined. In the case of apoptosis, fragmentation is performed by calcium activated DNAse (CAD), which cleaves the DNA between nucleosomes to generate a ladder pattern when evaluated in agarose gels (Figure 6; Bedoui et al., 2020). This enzyme is activated upon degradation of its inhibitor (ICAD) by caspase-3 (Figure 6). However, it is important to consider that other types of cell death, such as necrosis, also induce DNA fragmentation, although not in a periodic pattern like apoptosis. It is important to keep in mind that the TUNEL technique by itself cannot distinguish whether the fragmentation is in a periodic manner or the result of multiple fragments (i.e., apoptosis vs. necrosis). Therefore, it is highly recommended that several techniques are used to elucidate the correct manner of cell death (Kroemer et al., 2009; Banfalvi, 2017).

One important question is whether the loss in buoyancy promotes cell death or whether given some damage (e.g., oxidative stress, loss of DNA integrity) in the embryos, cell death is induced and results in a loss of buoyancy (Figure 6). The data in this study suggest that from embryos in the 2/4 cell state, there is a higher proportion that will go into cell death in embryos with low buoyancy. In other species, it has been observed that the beginning of physiological apoptosis coincides with the activation of the embryonic genome, which occurs in the blastula stage (Kane and Kimmel, 1993; O’Boyle et al., 2007; Fernández et al., 2013). Therefore, it is possible that the apoptosis markers observed from the blastula in both floating and low-buoyancy embryos correspond to molecules involved in physiological apoptosis. In the case of the 2/4 cell and morula stages, where a consistent increase in the expression of caspases and other markers of cell death was not observed, it is possible that cell death is due to a different mechanism that involves environmental damage or is due to a maternal effect where optimal conditions for the development and ovulation of good quality oocytes did not exist (Morais et al., 2012; Thomé et al., 2012). Therefore, embryos derived from these oocytes would present increased expression of markers of cell death and match those with low buoyancy.

In conclusion, we have presented for the first-time suggestive evidence that the loss of buoyancy in early embryos in the pelagic fish *S. lalandi* is related to an increase in apoptosis. However, we cannot rule out the participation of other mechanisms of cell death. To date, most of the information on early fish development comes from benthic embryo models (e.g., killifish, zebrafish), where the egg/embryo buoyancy is not an important characteristic in determining developmental potential. Further studies are required to determine the mechanisms at the molecular level that connect buoyancy with the activation of cell death.

## Data Availability Statement

The raw data supporting the conclusions of this article will be made available by the authors, without undue reservation.

## Ethics Statement

The animal study was reviewed and approved by Ethics and Animal Care Committees of Faculty of Biological Sciences of Pontifical Catholic University of Chile, Faculty of Veterinary Sciences, University of Chile. Written informed consent was obtained from the owners for the participation of their animals in this study.

## Author Contributions

MO and CG: performing experiments and collecting data. PD: collecting data and primer design. DP-G: manuscript writing and data analysis. RO: manuscript writing. RM: data analysis and manuscript writing. JP: experimental design planning, data analysis, and final manuscript approval. All authors contributed to the article and approved the submitted version.

## Conflict of Interest

The authors declare that the research was conducted in the absence of any commercial or financial relationships that could be construed as a potential conflict of interest.

## Footnotes

1 www-genome.wi.mit.edu/cgi-bin/primer/primer3.cgi

## References

[B1] Ådlandsvik B. Coombs S. Sundby S. Temple G. (2001). Buoyancy and vertical distribution of eggs and larvae of blue whiting (*Micromesistius poutassou*): observations and modelling. *Fish. Res.* 50 59–72. 10.1016/S0165-7836(00)00242-3

[B2] AnvariFar H. Amirkolaie A. K. Miandare H. K. Ouraji H. Jalali M. A. Üçüncü S. İ. (2017). Apoptosis in fish: environmental factors and programmed cell death. *Cell. Tissue. Res.* 368 425–439. 10.1007/s00441-016-2548-x 28035476

[B3] Banfalvi G. (2017). Methods to detect apoptotic cell death. *Apoptosis* 22 306–323. 10.1007/s10495-016-1333-3 28035493

[B4] Bedoui S. Herold M. J. Strasser A. (2020). Emerging connectivity of programmed cell death pathways and its physiological implications. *Nat. Rev. Mol. Cell Biol.* 21 678–695. 10.1038/s41580-020-0270-8 32873928

[B5] Bresciani E. Broadbridge E. Liu P. P. (2018). An efficient dissociation protocol for generation of single cell suspension from zebrafish embryos and larvae. *MethodsX* 5 1287–1290. 10.1016/j.mex.2018.10.009 30364607 PMC6197777

[B6] Carnevali O. Cionna C. Tosti L. Lubzens E. Maradonna F. (2006). Role of cathepsins in ovarian follicle growth and maturation. *Gen. Comp. Endocrinol.* 146 195–203. 10.1016/j.ygcen.2005.12.007 16430893

[B7] Carnevali O. Mosconi G. Cambi A. Ridolfi S. Zanuy S. Polzonetti-Magni A. M. (2001b). Changes of lysosomal enzyme activities in sea bass (*Dicentrarchus labrax*) eggs and developing embryos. *Aquaculture* 202 249–256. 10.1016/S0044-8486(01)00775-X

[B8] Carnevali O. Mosconi G. Cardinali M. Meiri I. Polzoneti-Magni A. M. (2001a). Molecular components related to egg viability in the gilthead sea bream, *Sparus aurata*. *Mol. Reprod. Dev.* 58 330–335. 10.1002/1098-2795(200103)58:3<330::AID-MRD11>3.0.CO;2-7 11170274

[B9] Carnevali O. Polzonetti V. Cardinali M. Pugnaloni A. Natalini P. Zmora N. (2003). Apoptosis in sea bream *Sparus aurata* eggs. *Mol. Reprod. Dev.* 66 291–296. 10.1002/mrd.10356 14502608

[B10] Chwieralski C. E. Welte T. Bühling F. (2006). Cathepsin-regulated apoptosis. *Apoptosis* 11 143–149. 10.1007/s10495-006-3486-y 16502253

[B11] Cole L. K. Ross L. S. (2001). Apoptosis in the developing zebrafish embryo. *Dev. Biol.* 240 123–142. 10.1006/dbio.2001.0432 11784051

[B12] Cullen S. Martin S. (2009). Caspase activation pathways: some recent progress. *Cell. Death. Differ.* 16 935–938. 10.1038/cdd.2009.59 19528949

[B13] D’Amelio M. Cavallucci V. Cecconi F. (2010). Neuronal caspase-3 signaling: not only cell death. *Cell. Death. Differ.* 17 1104–1114. 10.1038/cdd.2009.180 19960023

[B14] D’Arcy M. S. (2019). Cell death: a review of the major forms of apoptosis, necrosis and autophagy. *Cell Biol. Int.* 43 582–592. 10.1002/cbin.11137 30958602

[B15] Dewson G. Kluck R. M. (2009). Mechanisms by which Bak and Bax permeabilise mitochondria during apoptosis. *J. Cell Sci.* 122(Pt 16) 2801–2808. 19795525 10.1242/jcs.038166 PMC2736138

[B16] Dix M. M. Simon G. M. Cravatt B. F. (2008). Global mapping of the topography and magnitude of proteolytic events in apoptosis. *Cell* 134 679–691. 10.1016/j.cell.2008.06.038 18724940 PMC2597167

[B17] Eimon P. M. Kratz E. Varfolomeev E. Hymowitz S. G. Stern H. Zha J. (2006). Delineation of the cell-extrinsic apoptosis pathway in the zebrafish. *Cell Death Differ.* 13 1619–1630. 10.1038/sj.cdd.4402015 16888647

[B18] Eimon P. Ashkenazi A. (2010). The zebrafish as a model organism for the study of apoptosis. *Apoptosis* 15 331–349. 10.1007/s10495-009-0432-9 20033783

[B19] Fabra M. Raldúa D. A. Power P. M. T. Deen J. Cerdà J. (2005). Marine fish egg hydration is aquaporin mediated. *Science* 307:545. 10.1126/science.1106305 15681377

[B20] Fernández C. G. Roufidou C. Antonopoulou E. Sarropoulou E. (2013). Expression of developmental-stage-specific genes in the gilthead sea bream *Sparus aurata* L. *Mar. Biotechnol.* 15 313–320. 10.1007/s10126-012-9486-8 23053055

[B21] Ferrari L. Pistocchi A. Libera L. Boari N. Mortini P. Bellipanni G. (2014). FAS/FASL are dysregulated in chordoma and their loss-of-function impairs zebrafish notochord formation. *Oncotarget* 5 5712–5724. 10.18632/oncotarget.2145 25071022 PMC4170636

[B22] Huang Q. Deveraux Q. L. Maeda S. Salvesen G. S. Stennicke H. R. Hammock B. D. (2000). Evolutionary conservation of apoptosis mechanisms: lepidopteran and baculoviral inhibitor of apoptosis proteins are inhibitors of mammalian caspase-9. *Proc. Natl. Acad. Sci. U.S.A.* 97 1427–1432. 10.1073/pnas.97.4.1427 10677478 PMC26450

[B23] Jerez S. Samper M. Santamaría F. J. Villamandos J. E. Cejas J. R. Felipe B. C. (2006). Natural spawning of greater amberjack (*Seriola dumerili*) kept in captivity in the Canary Islands. *Aquaculture* 252 199–207. 10.1016/j.aquaculture.2005.06.031

[B24] Jung K. M. Folkvord A. Kjesbu O. Agnalt A. L. Thorsen A. Sundby S. (2012). Egg buoyancy variability in local populations of Atlantic cod (*Gadus morhua*). *Mar. Biol.* 159 1959–1980. 10.1007/s00227-012-1984-8 24391277 PMC3873015

[B25] Jung K. M. Folkvord A. Kjesbu O. S. Sundby S. (2014). Experimental parameterization of principal physics in buoyancy variations of marine teleost eggs. *PLoS One* 9:e104089. 10.1371/journal.pone.0104089 25122447 PMC4133173

[B26] Kane D. A. Kimmel C. B. (1993). The zebrafish midblastula transition. *Development.* 119 447–456. 8287796 10.1242/dev.119.2.447

[B27] Kiraz Y. Adan A. Kartal Yandim M. Baran Y. (2016). Major apoptotic mechanisms and genes involved in apoptosis. *Tumour. Biol.* 37 8471–8486. 10.1007/s13277-016-5035-9 27059734

[B28] Kroemer G. Galluzzi L. Vandenabeele P. Abrams J. Alnemri E. S. Baehrecke E. H. (2009). Classification of cell death: recommendations of the Nomenclature Committee on Cell Death 2009. *Cell Death Differ.* 16 3–11. 10.1038/cdd.2008.150 18846107 PMC2744427

[B29] Luthi A. U. Martin S. J. (2007). The CASBAH: a searchable database of caspase substrates. *Cell Death Differ.* 14 641–650. 10.1038/sj.cdd.4402103 17273173

[B30] Minarowska A. Minarowski L. Karwowska A. Gacko M. (2007). Regulatory role of cathepsin D in apoptosis. *Folia Histochem. Cytobiol.* 45 159–163. 17951163

[B31] Morais R. Thomé R. Santos H. Bazzoli N. Rizzo E. (2016). Relationship between bcl-2, bax, beclin-1, and cathepsin-D proteins during postovulatory follicular regression in fish ovary. *Theriogenology* 85 1118–1131. 10.1016/j.theriogenology.2015.11.024 26719039

[B32] Morais R. D. Thomé R. G. Lemos F. S. Bazzoli N. Rizzo E. (2012). Autophagy and apoptosis interplay during follicular atresia in fish ovary: a morphological and immunocytochemical study. *Cell. Tissue. Res.* 347 467–478. 10.1007/s00441-012-1327-6 22314847

[B33] Moran D. Smith C. Gara B. Poortenaar C. (2007). Reproductive behavior and early development in yellowtail kingfish (*Seriola lalandi* Valenciennes 1833). *Aquaculture* 262 95–104. 10.1016/j.aquaculture.2006.10.005

[B34] Moreno R. D. Urriola-Munoz P. Lagos-Cabre R. (2011). The emerging role of matrix metalloproteases of the ADAM family in male germ cell apoptosis. *Spermatogenesis* 1 195–208. 10.4161/spmg.1.3.17894 22319668 PMC3271662

[B35] O’Boyle S. Bree R. T. McLoughlin S. Grealy M. Byrnes L. (2007). Identification of zygotic genes expressed at the midblastula transition in zebrafish. *Biochem. Biophys. Res. Commun.* 358 462–468. 10.1016/j.bbrc.2007.04.116 17490614

[B36] Orellana J. Waller B. Wecker B. (2014). Culture of yellowtail kingfish (*Seriola lalandi*) in a marine recirculating aquaculture system (RAS) with artificial seawater. *Aquacult. Eng.* 58 20–28. 10.1016/j.aquaeng.2013.09.004

[B37] Ospina-Álvarez A. Palomera I. Parada C. (2012). Changes in egg buoyancy during development and its effects on the vertical distribution of anchovy eggs. *Fish. Res.* 117–118 86–95. 10.1016/j.fishres.2011.01.030

[B38] Palomino J. Herrera G. Dettleff P. Martínez V. (2014). Growth differentiation factor 9 and bone morphogenetic protein 15 expression in previtellogenic oocytes and during early embryonic development of yellow-tail kingfish *Seriola lalandi*. *Biol. Res.* 47:60. 10.1186/0717-6287-47-60 25723449 PMC4335437

[B39] Palomino J. Herrera G. Dettleff P. Patel A. Torres-Fuentes J. L. Martínez V. (2017). Assessment of cathepsin mRNA expression and enzymatic activity during early embryonic development in the yellowtail kingfish *Seriola lalandi*. *Anim. Reprod. Sci.* 80 23–29. 10.1016/j.anireprosci.2017.02.009 28262464

[B40] Pérez-Robles J. Diaz F. Re A. D. Giffard-Mena I. Abdo-de la Parra M. I. Ibarra-Castro L. (2015). Osmoregulation, growth, and survival during the larval development of bullseye puffer fish *Sphoeroides annulatus* (Jenyns, 1842, Pisces: Tetraodontidae). *Mar. Freshw. Behav. Phy.* 48 397–415. 10.1080/10236244.2015.1085692

[B41] Peter M. E. Barnhart B. C. Algeciras-Schimnich A. (2003). “CD95L/FasL and its receptor CD95 (APO-1/Fas),” in *The Cytokine Handbook*, 4th Edn, eds Thomson A. W. Lotze M. T. (London: Elsevier Science Ltd).

[B42] Riis-Vestergaard J. (1987). Physiology of teleost embryos related to environmental challenges. *Sarsia* 72 351–358. 10.1080/00364827.1987.10419735

[B43] Samaee S. M. Mente E. Estevez A. Gimenez G. Lahnsteiner F. (2010). Embryo and larva development in common dentex (*Dentex dentex*), a pelagophil teleost: the quantitative composition of egg-free amino acids and their interrelations. *Theriogenology* 73 909–919. 10.1016/j.theriogenology.2009.11.017 20083301

[B44] Sanchís-Benlloch P. J. Nocillado J. Ladisa C. (2017). *In-vitro* and *in vivo* biological activity of recombinant yellowtail kingfish (*Seriola lalandi*) follicle stimulating hormone. *Gen. Comp. Endocrinol.* 241 41–49. 10.1016/j.ygcen.2016.03.001 26965950

[B45] Sawaguchi S. Kagawa H. Ohkubo N. Hiramatsu N. Sullivan C. V. Matsubara T. (2006). Molecular characterization of three forms of vitellogenin and their yolk protein products during oocyte growth and maturation in red seabream (*Pagrus major*), a marine teleost spawning pelagic eggs. *Mol. Reprod. Dev.* 73 719–736. 10.1002/mrd.20446 16541459

[B46] Seoka M. Yamada S. Iwata Y. Yanagisawa T. Nakagawa T. Kumai H. (2003). Differences in the biochemical content of buoyant and non-buoyant eggs of the Japanese eel, Anguilla japonica. *Aquaculture* 216 355–362. 10.1016/S0044-8486(02)00459-3

[B47] Seyrek K. Lavrik I. N. (2019). Modulation of CD95-mediated signaling by post-translational modifications: towards understanding CD95 signaling networks. *Apoptosis* 24 385–394. 10.1007/s10495-019-01540-0 31069559

[B48] Shi Y. (2002). Apoptosome: the cellular engine for the activation of caspase-9. *Structure* 10 285–288. 10.1016/S0969-2126(02)00732-3 12005427

[B49] Shi Y. (2006). Mechanical aspects of apoptosome assembly. *Curr. Opin. Cell Biol.* 18 677–684. 10.1016/j.ceb.2006.09.006 17046227

[B50] Sokal R. R. (1995). *Biometry: The Principles and Practice of Statistics in Biological Research.* New York, NY: W. H. Freeman, 887.

[B51] Stuart K. R. Drawbridge M. A. (2013). Captive spawning and larval rearing of California yellowtail (*Seriola lalandi*). *Aquac. Res.* 44 728–737. 10.1111/j.1365-2109.2011.03077.x

[B52] Suda T. Takahashi T. Golstein P. Nagata S. (1993). Molecular cloning and expression of the fas ligand, a novel member of the tumor necrosis factor family. *Cell* 75 1169–1176. 10.1016/0092-8674(93)90326-l 7505205

[B53] Sundby S. Kristiansen T. (2015). The principles of buoyancy in marine fish eggs and their vertical distributions across the world oceans. *PLoS One* 10:e0138821. 10.1371/journal.pone.0138821 26465149 PMC4605736

[B54] Takahashi T. Tanaka M. Inazawa J. Abe T. Suda T. Nagata S. (1994). Human fas ligand: gene structure, chromosomal location and species specificity. *Int. Immunol.* 6 1567–1574. 10.1093/intimm/6.10.1567 7826947

[B55] Takle H. Andersen O. (2007). Caspases and apoptosis in fish. *J. Fish. Biol.* 71 326–349.

[B56] Thomé R. Domingos F. Santos H. Martinelli P. Sato Y. Rizzo E. (2012). Apoptosis, cell proliferation and vitellogenesis during the folliculogenesis and follicular growth in teleost fish. *Tissue Cell* 44 54–62. 10.1016/j.tice.2011.11.002 22153985

[B57] Tittel J. N. Steller H. (2000). A comparison of programmed cell death between species. *Genome Biol.* 1:REVIEWS0003. 10.1186/gb-2000-1-3-reviews0003 11178240 PMC138857

[B58] Valencia C. A. Bailey C. Liu R. (2007). Novel zebrafish caspase-3 substrates. *Biochem. Biophys. Res. Commun.* 361 311–316. 10.1016/j.bbrc.2007.06.173 17643392

[B59] Vandesompele J. De Preter K. Pattyn F. Poppe B. Van Roy N. De Paepe A. (2002). Accurate normalization of real-time quantitative RT-PCR data by geometric averaging of multiple internal control genes. *Genome Biol.* 3:research0034.1. 10.1186/gb-2002-3-7-research0034 12184808 PMC126239

[B60] Willis S. N. Adams J. M. (2005). Life in the balance: how BH3-only proteins induce apoptosis. *Curr. Opin. Cell. Biol.* 17 617–625. 10.1016/j.ceb.2005.10.001 16243507 PMC2930980

[B61] Yamashita M. Mizusawa N. Hojo M. Yabu T. (2008). Extensive apoptosis and abnormal morphogenesis in pro-caspase-3 transgenic zebrafish during development. *J. Exp. Biol.* 211 1874–1881. 10.1242/jeb.012690 18515717

[B62] Yang S. G. Hur S. W. Ji S. C. (2016). Morphological development of embryo, larvae and juvenile in yellowtail kingfish, *Seriola lalandi*. *Dev. Reprod.* 20 131–140. 10.12717/DR.2016.20.2.131 27660828 PMC5027218

[B63] Youle R. J. Strasser A. (2008). The BCL-2 protein family: opposing activities that mediate cell death. *Nat. Rev. Mol. Cell Biol.* 9 47–59. 10.1038/nrm2308 18097445

